# CircRNA Sequencing Analysis Reveals the Regulatory Role of circ_CDR1as in Penile Squamous Cell Carcinoma via the ceRNA Network

**DOI:** 10.7150/ijms.112109

**Published:** 2025-06-12

**Authors:** Lin Du, Shanshan Lv, Zhenzhen Jiang, Xiaohong Song, Leping Liu, Rong Huang, Xinmin Nie, Rong Gui, Jian Li, Junhua Zhang, Jie Guo, Jian Cao, Zhizhong Liu, Zhaojian Gong, Yanwei Luo

**Affiliations:** 1 Department of Blood Transfusion, The Third Xiangya Hospital, Central South University, Changsha 410013, Hunan, China.; 2 Department of Pediatrics, The Third Xiangya Hospital, Central South University, Changsha 410013, Hunan, China.; 3 Department of Laboratory Medicine, The Third Xiangya Hospital, Central South University, Changsha 410013, Hunan, China.; 4 China National Clinical Research Center for Geriatric Disorders, National Institution of Drug Clinical Trial, Xiangya Hospital, Central South University, Changsha 410008, Hunan, China.; 5 Department of Urology, Hunan Cancer Hospital, The Affiliated Cancer Hospital of Xiangya School of Medicine, Central South University, Changsha 410013, Hunan, China.; 6 Department of Oral and Maxillofacial Surgery, The Second Xiangya Hospital of Central South University, Changsha 410011, Hunan, China.

**Keywords:** penile squamous cell carcinoma, ceRNA network, circRNA, circ_CDR1as, GATA6

## Abstract

Penile squamous cell carcinoma (PSCC) is a malignant reproductive tumor, and circRNAs are essential regulators in the progression of cancers. However, their specific roles in PSCC have not been adequately investigated, which are available to function via the competing endogenous RNA (ceRNA) network. We collected 5 normal and 6 PSCC tissue samples for bulk RNA sequencing and obtained differentially expressed circRNAs (DE-circRNAs) and differentially expressed mRNAs (DE-mRNAs). Then we conducted a correlation analysis between them, followed by the prediction and intersection of their target miRNAs and mRNAs by online databases. We constructed a ceRNA network of 11 circRNAs, 14 miRNAs, and 33 mRNAs. The enrichment analysis showed that the positively circRNA-related mRNAs are mainly involved in the positive regulation of leukocyte activation and cell adhesion. It was revealed that circ_CDR1as and its target gene GATA6 were significantly downregulated in PSCC, and their reduction in tumor samples was validated by an external dataset and qRT-PCR, indicating a potential regulatory relationship between them. Our study identified that circ_CDR1as may affect PSCC progression through the circRNA-miRNA-mRNA network, and GATA6 could be one of its possible targets for PSCC therapy strategies.

## Introduction

Penile cancer is a relatively rare reproductive malignancy that includes sarcoma, melanoma, basal cell carcinoma, and squamous cell carcinoma. Clinically, about 95% of cases were classified as squamous cell carcinoma^1^. Penile squamous cell carcinoma (PSCC) is most frequent in developing nations such as Southern Africa, South Asia, and South America, but it is rare in developed nations, with a frequency of roughly one in 100,000 men^2^. However, current statistics indicate that the incidence of PSCC is rising in several countries^3^-^5^. PSCC can be caused by a variety of variables, such as immunological factors, chronic inflammation, smoking, circumcision, and HPV infection, which is one of the main causes of the disease's elevated risk^6^, ^7^. The increase in HPV infection can partially explain the rise in the incidence of PSCC in recent years, and HPV ravages are also one of the key causes for the higher incidence rates of PSCC in regions such as Southern Africa and South America^8^. Actually, routine HPV vaccination in men and childhood circumcision can help minimize the prevalence of squamous penile cancer^9^, ^10^.

In PSCC, the groin is the key site of lymphatic spread, while early diagnosis and early surgical staging in the groin can significantly improve patient outcomes^6^, ^11^. For advanced PSCC, cisplatin-based chemotherapy is the standard method of treatment, but it has very poor responsiveness and high mortality^12^, ^13^. Since the pathophysiology and genetics of PSCC are poorly understood nowadays, genetic research can help bring fresh insights into developing more effective treatments.

In the human body, only 1%-2% of RNA are protein-coding mRNAs^14^, in addition, there are also a large number of non-coding RNAs (ncRNAs), such as microRNAs (miRNAs), long non-coding RNAs (lncRNAs), and circular RNAs (circRNAs)^15^, which has triggered the interest of researchers. By binding to miRNA response elements (MREs) on mRNAs, miRNAs have been shown to control gene expression at the post-transcriptional level^16^. However, some circRNAs, lncRNAs, and pseudogenes share the same MREs as mRNAs, allowing them to compete for miRNA binding and thus indirectly regulate mRNA expression. This competition results in the formation of a complex regulatory network known as competing endogenous RNA (ceRNA)^17^. The ceRNA network is involved in tumor cell proliferation, metastasis, angiogenesis, chemotherapy resistance, and tumor immunity^18^. For example, it has been shown that circRPPH1 acts as a molecular sponge for miR-326, controls ITGA5 expression, and activates the FAK/PI3K/AKT pathway to promote the progression of triple-negative breast cancer (TNBC)^19^; The circ-0064516 exerts oncogenic effects by enhancing the migration and angiogenesis of cervical cancer cells through the miR-6805-3p/MAPK1 axis^20^; LINC01607 acts as a ceRNA for mirRNA-892b, which enhances the resistance of advanced hepatocellular carcinoma patients to the standard therapeutic drug lenvatinib by up-regulating P62 and decreasing the ROS level^21^; And circRNF216 can act as ceRNA for miR-576-5p, attenuating the inhibition of miR-576-5p on its target ZC3H12C, which will upregulate and then increase the infiltration level of CD8+ T-cells, and ultimately inhibiting the development of colorectal cancer (CRC)^22^.

Currently, only a few studies have focused on the mechanism of the ceRNA network in PSCC. However, the role of circRNAs and their potential target molecules in PSCC remains uncertain. Therefore, we performed circRNA-seq analysis on PSCC samples to identify differentially expressed circRNAs and predict their complicated regulatory connections with miRNAs and mRNAs to investigate new targets for PSCC therapy.

## Materials and Methods

### Sample collection

We collected 5 normal penile tissues and 6 PSCC tissues from the Department of Urology, Hunan Cancer Hospital, which were sent for circRNA sequencing and mRNA sequencing to obtain expression profile data. An additional 11 normal penile tissues and 11 PSCC tissues were collected for validation in subsequent experiments. The tissues of participants are fresh and decent for sequencing. After the tissue samples were collected in the operating room, the blood stain was quickly washed off with PBS and then dried with absorbent paper before being placed in -80℃ for freezing. The study process was reviewed and approved by the Ethics Committee of Hunan Cancer Hospital (KYJJ-2020-071), and all patients received informed consent, according to the principles of the Declaration of Helsinki. The clinical information of all patients is provided in Table 1.

### CircRNA sequencing

The whole process of this study was following the principles of the Declaration of Helsinki. Firstly, the total RNA was extracted and rRNAs were removed. The enriched mRNAs and ncRNAs were fragmented and reverse-transcribed into cDNA. Second-strand cDNA was synthesized, and cDNA fragments were ligated to Illumina sequencing adapters, followed by the steps of Uracil-N-Glycosylase treatment, PCR amplification, and sequencing using IlluminaHiSeqTM 4000 by Gene Denovo Biotechnology Co. (Guangzhou, China). The offline raw reads were processed using fastp (version 0.18.0)^23^ to filter the low-quality data and obtain HQ Clean Reads. The parameters were as follows: removing reads containing adapters and reads containing more than 10% of unknown nucleotides, and more than 50% of low-quality (Q-value ≤ 20) bases. And then they were compared with the reference genome for each sample separately by HISAT2 (version 2.1.1)^24^. The comparison results were submitted to CIRI2 software to identify the circRNAs, which were analyzed in three main parts: circRNA statistics, expression calculation, and database annotation, including circBank (http://www.circbank.cn/) and circBase (http://circbase.org/) database annotation of circRNAs, where annotated circRNAs were defined as known circRNAs and unannotated circRNAs were defined as novel circRNAs.

### Bioinformatics analysis

Firstly, GO and KEGG enrichment analyses were performed on all circRNA source genes that had been identified in all samples, demonstrating an overall profile of the major pathways involved. Then, the gene expression profiles of the samples were differentially analyzed via the DESeq2 package in R (|log_2_FC| > 2, *p*.adj < 0.001) to obtain differentially expressed circRNAs (DE-circRNAs) and differentially expressed mRNAs (DE-mRNAs), and a correlation analysis was conducted between them using the cor.test function based on a preset threshold (|r| > 0.7, *p*.adj < 0.001). We applied Pearson correlation analysis to obtain positively and negatively correlated circRNA-mRNA pairs with their correlation coefficients and *p* values. We also conducted functional enrichment analyses for positively circRNA-related mRNAs. This process utilizes the clusterProfiler package in R.

### Construction of the ceRNA network

For known DE-circRNAs in the positive correlation section, we used the starBase (https://rnasysu.com/encori/), circBank (http://www.circbank.cn/), and Circular RNA Interactome (https://circinteractome.nia.nih.gov/) websites to predict the target miRNAs of the known circRNAs, which were analyzed by miRDB (https://mirdb.org/), miRTarBase (https://mirtarbase.cuhk.edu.cn/~miRTarBase/), and TargetScan (https://www.targetscan.org/) websites to predict the target mRNAs of the filtered miRNAs, and the latter was taken as intersections with the positively circRNA-related mRNAs, and finally, Cytoscape was applied to construct the circRNA-miRNA-mRNA ceRNA network. The mRNAs in the ceRNA network were analyzed by GO enrichment, and GSE196978 was used to verify the differential expression of mRNAs targeted by circ_CDR1as. In addition, Pearson correlation analysis was carried out for all circRNAs and mRNAs in the ceRNA network.

### RNA extraction

The total RNA was extracted from tissue samples using Trizol Reagent (15596018CN, Invitrogen, USA). First, chloroform was added to tissue samples, mixed well, and centrifuged at 4°C and 12,000 rpm for 15 min. Transfer the upper aqueous phase to another centrifuge tube and add an equal volume of isopropanol. After shaking and centrifugation again, discard the supernatant. The RNA precipitation was washed twice with 75% ethanol prepared with DEPC water, centrifuged at 7,500 rpm at 4 °C for 5 min, and after the supernatant was removed, it was dried at room temperature. Finally, RNA is dissolved in DEPC water, and the concentration and purity of each RNA sample are determined by a NanoDrop®ND-1000 spectrophotometer (Thermo Fisher, USA). All experimental procedures were performed on ice.

### Quantitative real-time polymerase chain reaction (qRT-PCR)

We synthesized cDNA from 1 μg of RNA using the Transcriptor First Strand cDNA Synthesis Kit (4897030001, Roche, USA). The qPCR amplification was performed using the SYBR Green qPCR Mix (RK21219, ABclonal, China). The 2 ul of cDNA, 2 ul of primers, 5 ul of SYBR Green qPCR Mix, and 1 ul of water were added to each well. A real-time PCR system (LightCycler480, Roche, USA) was used to detect the relative expression of genes by the 2^-ΔΔCt^ method and the number of amplified products in each PCR cycle was measured in real-time by fluorescence signal intensity. RNU6 and GAPDH were the internal reference genes of circRNA and mRNA for normalization, respectively. The synthesized primer sequences (Sangon Biotech, China) are as follows: circ_CDR1as: Forward: 5'-TCTGCTCGTCTTCCAACATC-3', Reverse: 5'-CGGAAACCCTGGATATTGCA-3'. GATA6: Forward: 5'-CTCAGTTCCTACGCTTCGCAT-3', Reverse 5'-GTCGAGGTCAGTGAACAGCA-3'. RNU6: Forward: 5'-CTCGCTTCGGCAGCACATATACT-3', Reverse: 5'-ACGCTTCACGAATTTGCGTGTC-3'. β-actin: Forward: GTCATTCCAAATATGAGATGCGT, Reverse: GCTATCACCTCCCCTGTGTG.

### Statistical analysis

All statistical analyses were performed using R software (version 4.2.2). Normally distributed data and non-normally distributed data were compared for differences between the two groups using the student's t-test and the Mann-Whitney U-test, respectively. Pearson correlation analysis was used to compare the correlation intensity between genes. The *p* value < 0.05 was considered statistically significant. The Benjamini-Hochberg method was used to adjust *p* values and control the false discovery rate (FDR) in the differential expression analysis.

## Results

### CircRNA-seq shows the landscape of circRNA distribution in samples

**Figure 1:**
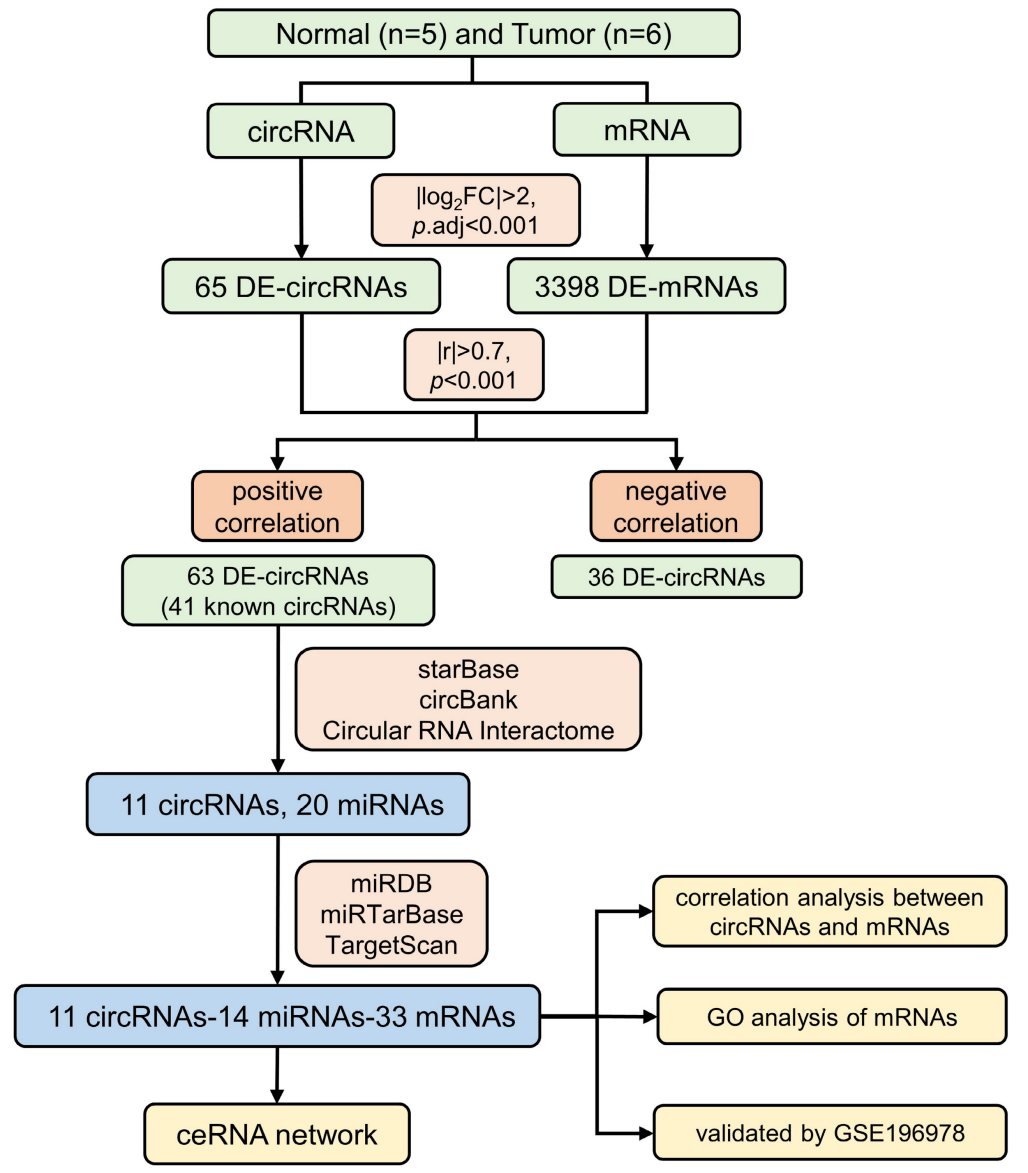
The flow chart of this study.

**Figure 2:**
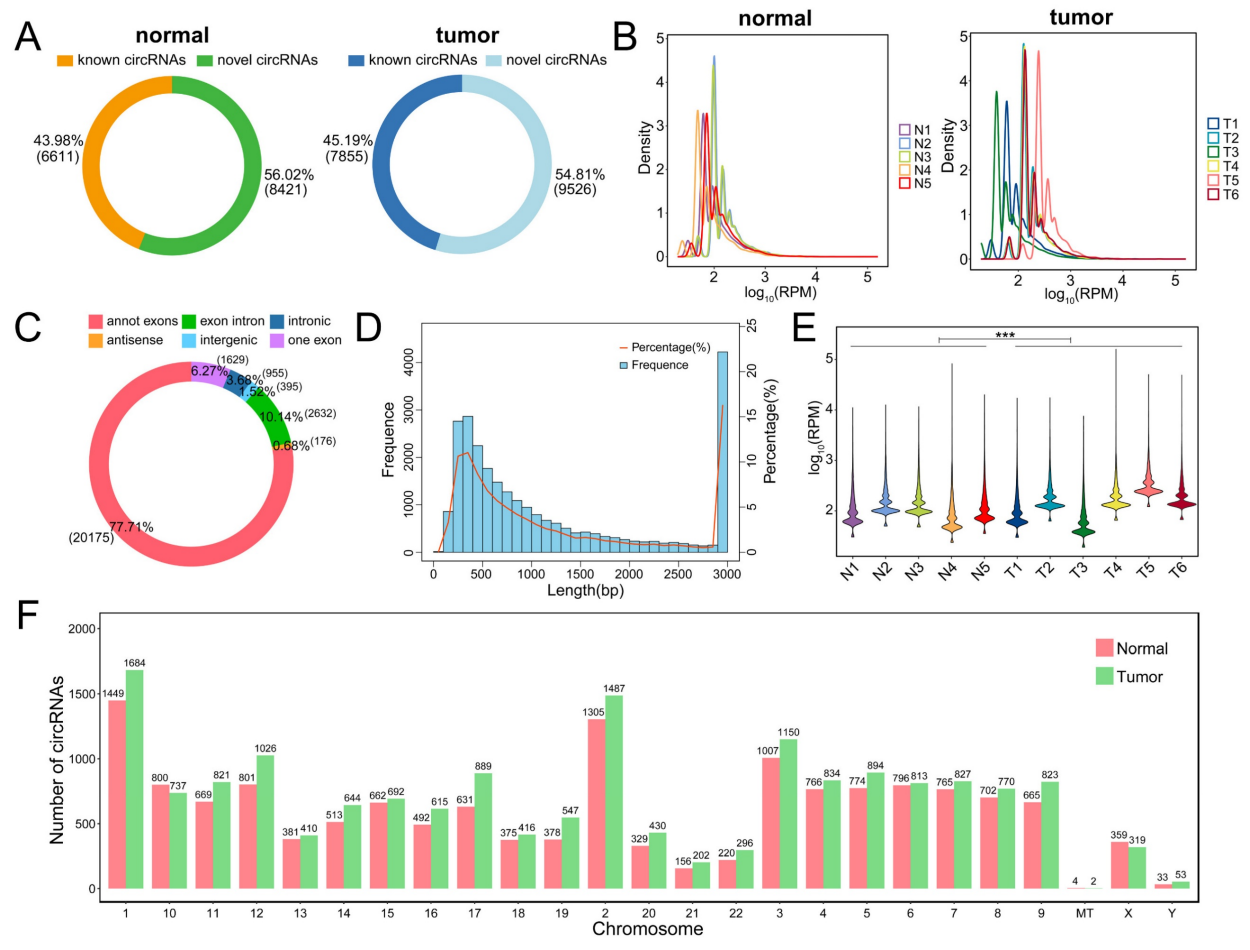
Overview of circRNA sequencing results. (A) Circle plots of known circRNAs and novel circRNAs in the normal and tumor groups. (B) Density plots of circRNA based on RPM values for each sample in the normal and tumor groups. (C) Circle plot of the identified circRNA type proportion. (D) Histogram and line plot of circRNA length distribution. (E) Violin plot of circRNA based on RPM values for each sample. (F) Barplot of circRNA chromosome distribution.

Five normal and six PSCC tissues collected from Hunan Cancer Hospital were sent for circRNA-seq, which yielded 20,391 mRNAs and 25,962 circRNAs, including 10,049 known circRNAs and 15,913 novel circRNAs. Detailed information on all identified circRNAs and the raw data expression profiles can be viewed in Table S1 and Table S2, respectively. The flowchart for this study is displayed in Figure 1. For all identified circRNAs, the tumor and normal groups contained similar proportions of known circRNAs, both slightly lower than the novel circRNAs (Figure 2A). Compared with the PSCC group, the abundance of circRNAs in the normal group was relatively concentrated, with peaks mainly focused on RPM (Reads Per Million mapped reads) values around 100 (Figure 2B). Among all circRNA types, annot exon had the highest percentage, exon intron followed, and antisense had the least (Figure 2C). Meanwhile, the length distribution plot of circRNAs showed that the percentage and frequency of circRNAs with lengths around 300 bp and 3000 bp were the highest, while between 400 bp and 2800 bp, their proportion gradually decreased with the extension of length (Figure 2D). We compared the expression profiles of circRNA in each sample and demonstrated that the expression of RPM values in the PSCC group was significantly higher than that in the normal group (Figure 2E). In addition, we can see the distribution of circRNA quantity in each chromosome (Figure 2F).

### Functional enrichment analysis of all circRNA source genes

**Figure 3:**
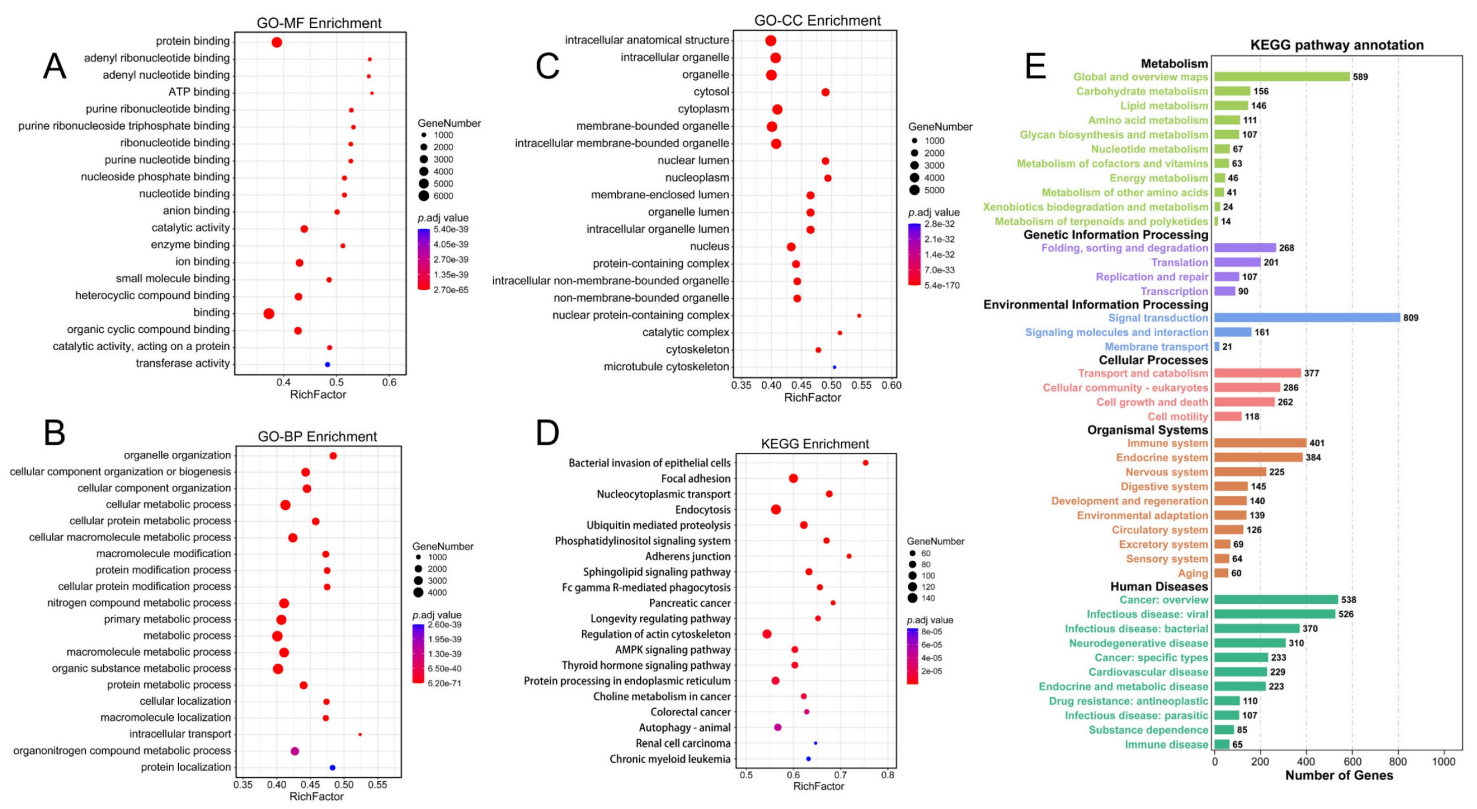
Functional enrichment analysis of all identified circRNAs. (A) GO-BP enrichment analysis of all circRNAs. (B) GO-BP enrichment analysis of all circRNAs. (C) GO-CC enrichment analysis of all circRNAs. (D) KEGG enrichment analysis of all circRNAs. (E) KEGG pathway annotation of all circRNAs.

After completing circRNA-seq, we conducted a functional enrichment analysis of all circRNA source genes to explore the biological functions and signaling pathways of circRNAs in PSCC. GO-MF enrichment analysis exhibited that circRNAs were mainly enriched in protein binding (Figure 3A). GO-BP enrichment analysis indicated that circRNAs were primarily involved in macromolecule metabolism, protein metabolism, and cellular metabolism (Figure 3B). GO-CC enrichment analysis suggested that circRNAs may participate in the formation of intracellular structures, organelles, and cytoplasm (Figure 3C). The results of the KEGG enrichment analysis were mainly related to endocytosis, focal adhesion, and regulation of the actin cytoskeleton (Figure 3D). The most enriched KEGG pathway annotations were folding, sorting and degradation, signal transduction, transport and catabolism, immune system, and cancer (Figure 3E). In conclusion, we can find that these circRNAs may play potential functions in protein binding, various metabolic processes, and endocytosis in PSCC progression.

### Differential analysis of circRNAs and enrichment analysis of positively circRNA-related mRNAs

**Figure 4:**
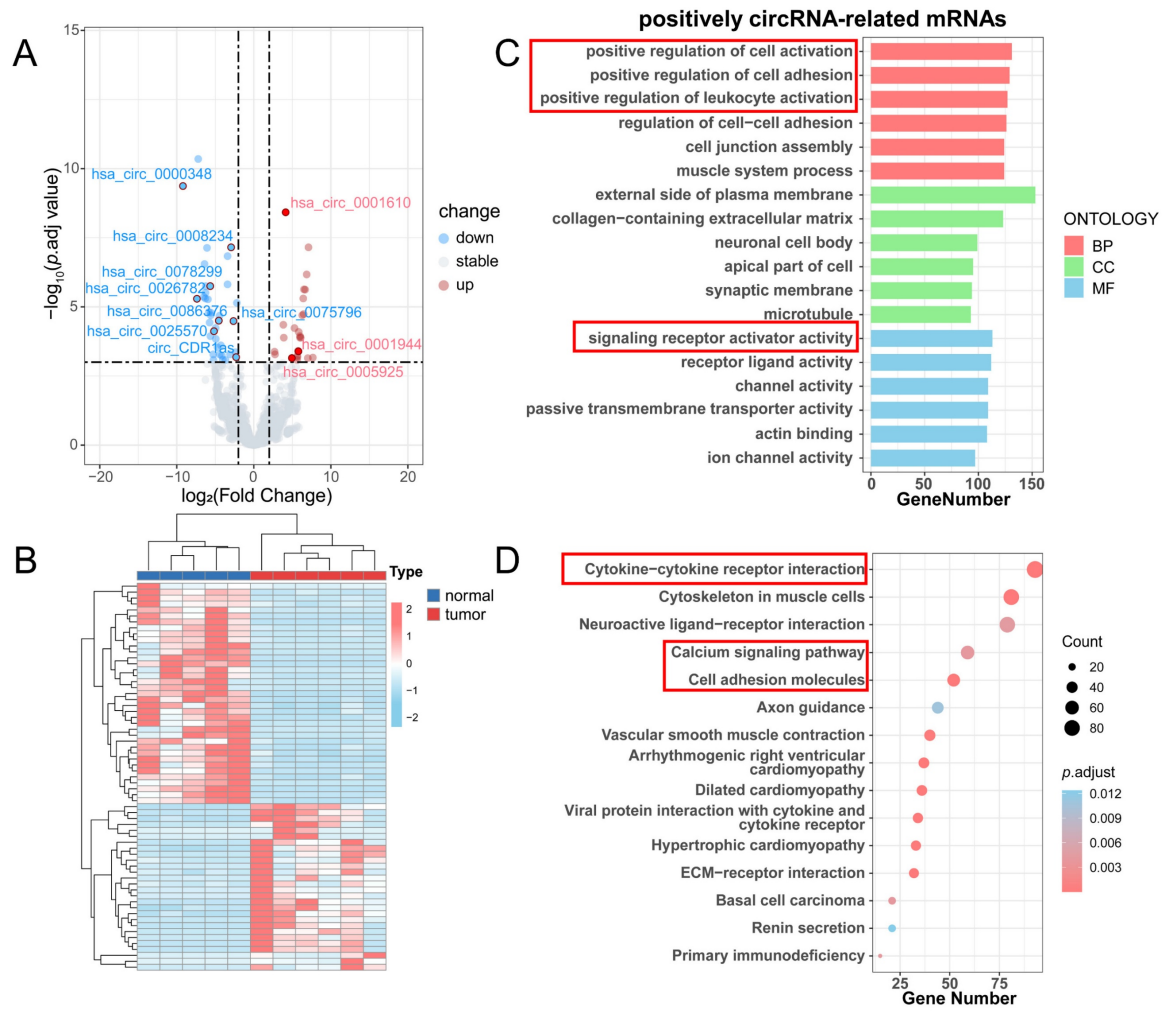
Differential analysis of circRNAs and enrichment analysis of positively circRNA-related mRNAs. (A) Volcano plot for differential analysis of circRNAs. (B) Heatmap for differential analysis of circRNAs. (C) GO enrichment analysis of positively circRNA-related mRNAs. (D) KEGG enrichment analysis of positively circRNA-related mRNAs.

We conducted a differential analysis of circRNAs and mRNAs according to the threshold (|log2FC| > 2, *p*.adj < 0.001), resulting in 65 differentially expressed circRNAs (DE-circRNAs) and 3398 differentially expressed mRNAs (DE-mRNAs) (Table S3). The volcano plot displayed 28 upregulated and 37 downregulated DE circRNAs (Figure 4A), and the heatmap demonstrated the overall DE-circRNA expression in the tumor and normal groups (Figure 4B). Subsequently, correlation analysis was implemented (|r| > 0.7, *p*.adj < 0.001) between DE-circRNAs and DE-mRNAs, in which it was obtained that 63 circRNAs were positively correlated with 3036 mRNAs and 36 circRNAs were negatively correlated with 696 mRNAs. Since circRNAs in the ceRNA network promote mRNA transcriptional activity by inhibiting the silence of target genes by miRNAs, the expression level of circRNAs is supposed to be positively associated with mRNAs. Therefore, a functional enrichment analysis of circRNA-related mRNAs was completed. GO enrichment analysis showed that these mRNAs mainly played roles in positive regulation of leukocyte activation, positive regulation of cell adhesion, and signaling receptor activator activities (Figure 4C). KEGG enrichment analysis exhibited that these mRNAs were enriched in cytokine-cytokine receptor interaction, the calcium signaling pathway, and cell adhesion molecules (Figure 4D).

### Construction of a ceRNA network containing DE-circRNAs and DE-mRNAs

**Figure 5:**
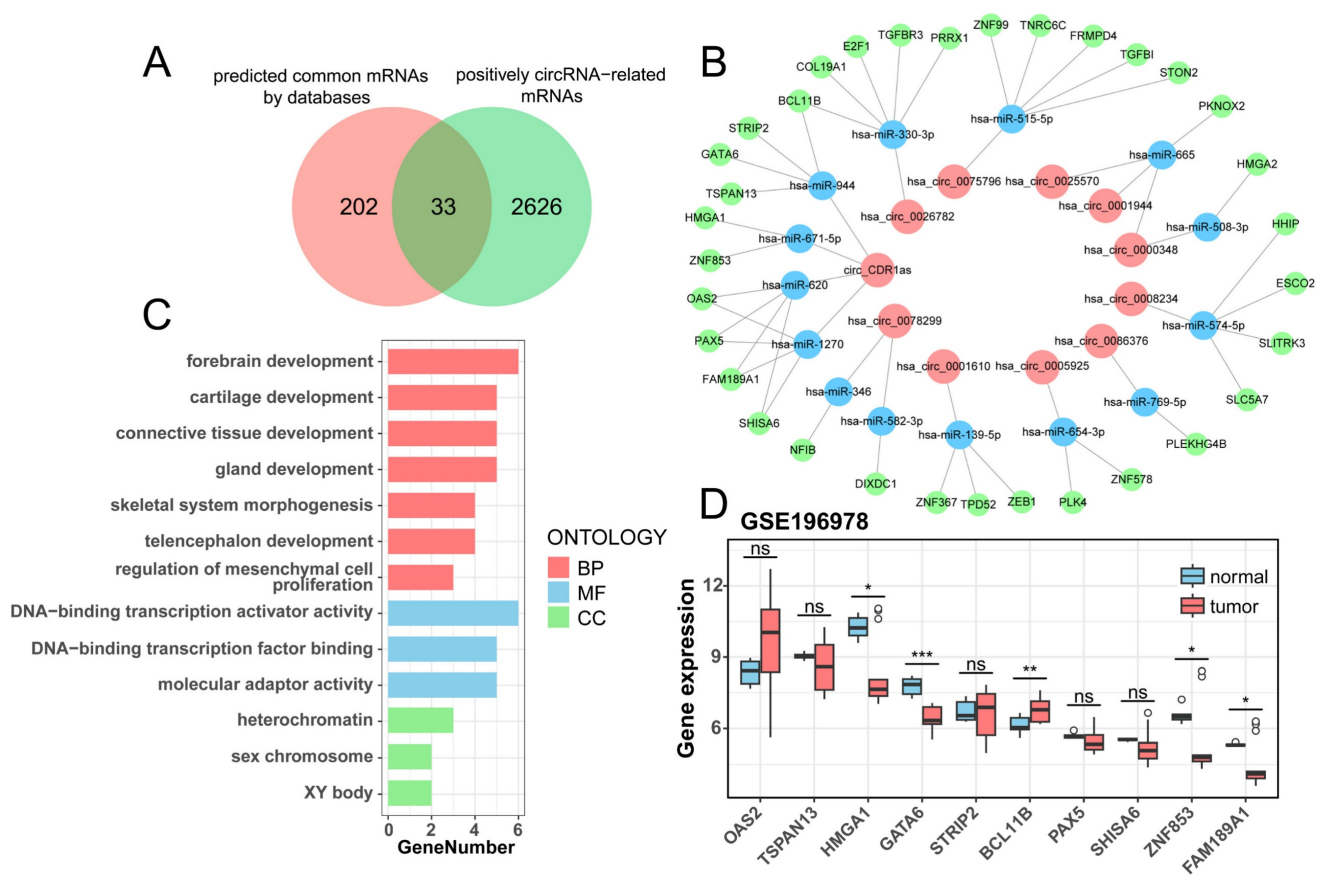
Construction of the ceRNA network. (A) The Venn plot suggests the intersection of predicted common mRNAs by databases and positively circRNA-related mRNAs. (B) The ceRNA network contains 11 circRNAs, 14 miRNAs, and 33 mRNAs. (C) GO enrichment analysis of mRNAs from the ceRNA network. (D) Boxplot of differential expression of circ_CDR1as target mRNAs validated by GSE196978.

There are 41 known DE-circRNAs out of 63 DE-circRNAs in the positive correlation section, put them into the starBase, circBank, and Circular RNA Interactome websites to get the target miRNAs predicted by circRNAs, and take the intersection to get 11 circRNAs targeting 20 miRNAs (Table 2). Subsequently, the target mRNAs were predicted by miRDB, miRTarBase, and TargetScan websites, and the common mRNAs were intersected with mRNAs which were positively correlated to the 11 circRNAs (r > 0.7, *p* < 0.05) to obtain miRNA and mRNAs (Table 3), ultimately constructing a ceRNA regulatory network of 11 circRNAs, 14 miRNAs, and 33 mRNAs (Figure 5B, Table S4). The Venn plot illustrates the process of taking intersections of predicted mRNAs by databases and filtered mRNAs by correlation analysis, as well as all mRNAs in the ceRNAs (Figure 5A). We analyzed the enrichment of 33 mRNAs in the ceRNA network and found that they were mainly enriched in forebrain development, chondrogenesis, and DNA-binding transcriptional activation activity (Figure 5C). We validated the differential expression of 10 mRNAs targeted by circ_CDR1as using an external dataset, GSE196978, and showed that GATA6, ZNF853, and FAM189A1 were significantly downregulated in the PSCC patients, while BCL11B had a clear overexpression in tumors (Figure 5D), which were consistent with those demonstrated by the sequencing analysis in this study.

### Correlation analysis between circRNAs and mRNAs in the ceRNA network

**Figure 6:**
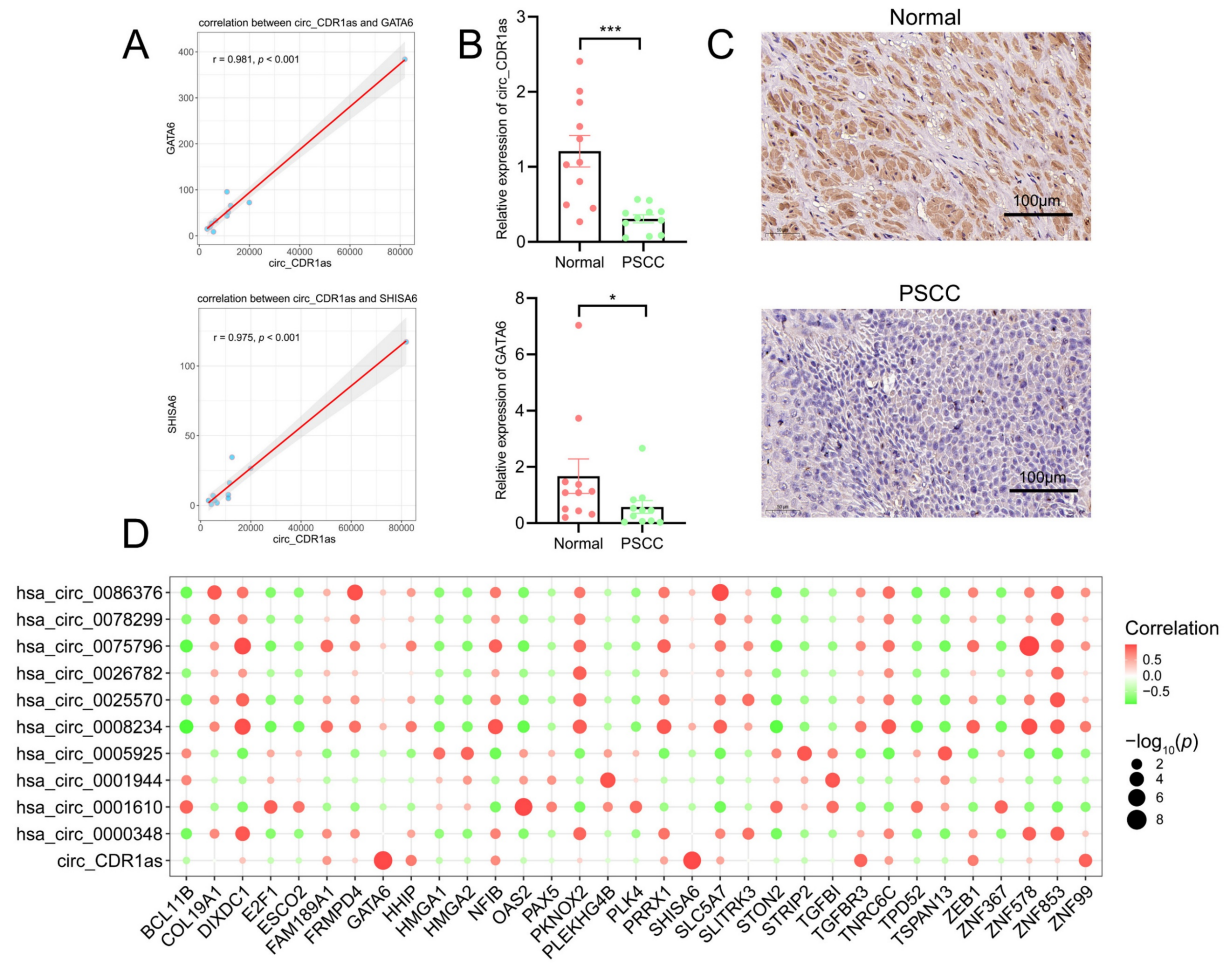
Correlation analysis of circRNAs and mRNAs from the ceRNA network. (A) Scatterplots suggest the correlation of circ_CDR1as with GATA6 and SHISA6. (B) The comparison of relative expressions of circ_CDR1as and GATA6 in normal and PSCC groups. The error bars in this graph represent the mean±SEM.^ ***^ *p* < 0.001, ^**^ *p* < 0.01, ^*^ *p* < 0.05. (C) The immunohistochemical results of GATA6 in normal and PSCC samples. (D) Bubble plot of the correlation of all circRNAs with all mRNAs from the ceRNA network.

Since the most abundant miRNAs and mRNAs were targeted by circ_CDR1as in the ceRNA network, we carried out an expression correlation analysis of 10 target mRNAs of circ_CDR1as. The results indicated that only two of them had positive correlations with circ_CDR1as (Figure 6A, Table 4), which were GATA6 (r =0.981, *p* < 0.001) and SHISA6 (0.975, *p* < 0.001). It was then verified by qRT-PCR that the expression levels of circ_CDR1as and GATA6 were significantly downregulated in PSCC patients compared with normal penile tissues, whereas SHISA6 did not change apparently (Figure 6B), which was consistent with the validation of GSE196978. The immunohistochemistry of GATA6 in normal and PSCC samples also showed its apparent downregulation in tumors (Figure 6C). Besides, HHIP (r = 0.753, *p* = 0.008), TGFBR3 (r = 0.864,* p* < 0.001), ZEB1 (r = 0.746, *p* = 0.008), ZNF99 (r = 0.871, *p* < 0.001), and NFIB (r = 0.661, *p* = 0.027) also showed favorable correlations with circ_CDR1as expression both in normal and PSCC tissues (Table S5). The 11 circRNAs and their highly related top mRNAs in the ceRNA network are shown in Table S6. Meanwhile, we implemented correlation analysis on all circRNAs and mRNAs within the ceRNA network, and it revealed the connection of circ_CDR1as with GATA6 and SHISA6 expression as well (Figure 6D).

## Discussion

Penile squamous cell carcinoma (PSCC), as a major medical burden for men's health worldwide, has gained relatively little attention compared with other cancers because it is rare in developed countries^6^. At the same time, it suffers from a lack of research funding, expertise, and medical personnel in developing regions^25^. Lymph node status is the most significant prognostic factor in PSCC despite various biomarkers that have been explored before^26^. In the 2022 WHO classification of urogenital system tumors, PSCC was categorized into HPV-associated and HPV-independent types based on block-type P16 positivity in immunohistochemistry (IHC)^27^. Our study collected 6 cases of PSCC tumor tissues and 5 cases of adjacent tissues for sequencing analysis. Patients with p16-positive status are considered HPV-positive, while those with p16-negative status are deemed HPV-negative. For advanced PSCC patients, the use of immunotherapies such as immune checkpoint blockade and HPV-directed vaccines is increasingly common, in addition to a multimodal treatment approach combining cisplatin-based chemotherapy and consolidating surgical treatments^28^. On the other hand, it is well known that circRNAs act as sponges for miRNAs and indirectly regulate the expression of mRNAs, exerting their functions as ceRNAs and thereby inducing or inhibiting multiple tumor development^29^. For example, it was shown that circRNA ciRS-7/circ_CDR1as mediates miR-1299 to target MMPs to maintain the metastatic phenotype of triple-negative breast cancer^30^. Another study indicated that circPDIA4 induced gastric cancer progression by enhancing MAPK pathway activity and promoting oncogenic circRNA biogenesis^31^. In other squamous cell carcinomas, circRNAs can play a regulatory role. Zhang LX *et al.* found that circHMGB2 relieves downstream CARM5 inhibition by sponging miR-1a-181p, which drives immunosuppression in lung squamous cell carcinoma^32^. However, few studies of circRNA in PSCC progression have been reported so far among the many.

Circ_CDR1as is transcribed from the cerebellar degeneration-related protein 1 (CDR1) locus on Xq27.1^33^. Previous studies have demonstrated that circ_CDR1as plays different roles in different kinds of tumors. We discovered that the expression of circ_CDR1as and its downstream target gene GATA6 was decreased in PSCC samples, but its other predicted target gene, SHISA6, did not show corresponding changes in expression levels in tissues. On the one hand, circ_CDR1as performs a pro-carcinogenic effect and enhances chemoresistance in certain cancers. For example, in oral squamous cell carcinoma (OSCC), circ_CDR1as can act as a sponge for miR-7, leading to the attenuation of the miR-7 targets RAF-1 and PIK3CD, which promotes metastatic progression of tumors by regulating the MAPK/AKT signaling pathway^34^. The circ_CDR1as can also enhance resistance to cisplatin-based chemotherapeutic drugs in non-small cell lung cancer (NSCLC) by targeting the miR-641/HOXA9 axis^35^. On the other hand, circ_CDR1as can resist tumor growth in some other cancers. For example, in ovarian cancer, circ_CDR1as acts as a sponge for miR-135b-5p, reducing cancer growth by suppressing the inhibitory effect of miR-135b-5p on hypoxia-inducible factor 1-alpha inhibitor^36^. In several investigations, circ_CDR1as has been shown to play a similar tumor-resistant effect in bladder cancer and glioblastoma^33^, ^37^. We discovered that circ_CDR1as expression in tumor tissues was lower than in normal tissues after collecting tumor and normal tissues from PSCC patients and performing circ-RNA sequencing. As a result, it is possible that circ_CDR1as has an oncogenic role in PSCC. Further research into its mechanism of action revealed that circ_CDR1as may function as a molecular sponge for miR-944 and miR-1270 in PSCC. It has also been shown that circ_CDR1as enhances the proliferation, migration, and invasion of hepatocellular carcinoma (HCC) via the miR-944/NOX4 pathway^38^, and as above described, to inhibit cisplatin resistance in ovarian cancer via the miR-1270/SCAI pathway. This finding indicates that restoring circ_CDR1as expression in PSCC may represent a novel therapeutic strategy for cancer treatment. HPV infection can affect the tumor immune microenvironment. Research evidence indicates that HPV-positive PSCC is more sensitive to radiotherapy and chemotherapy treatment^39^. We conducted a differential analysis of the expression of circ_CDR1as in the tissues of 2 HPV-positive and 3 HPV-negative PSCC patients and found no significant difference between the two groups (*p*.adj = 0.8). However, due to the limitation of the small number of cases in this study, it is temporarily impossible to determine whether there is a correlation between the expression of circ_CDR1as and HPV infection.

The mechanism of miRNA action in cancers has been thoroughly researched, but only a limited study has been conducted in PSCC. The expression of miR-218 in PSCC is lower than in normal tissues, and its epigenetic silencing is a common feature of HR-HPV(+) and HR-HPV(-) PSCCs without P53 immunohistochemical staining^40^. HOXD11 acts as a kind of tumor suppressor, while miR-138-5p can promote tumor malignant progression in PSCC by binding to the 3' non translated region to inhibit HOXD11 post-transcriptional translation and promote malignant tumor progression^41^. Currently, there are very few studies investigating miR-944 in PSCC, and its functional role remains largely unexplored, but miR-944 plays a key role in other cancers. For example, miR-944 regulates the progression of OSCC by modulating CDH2 expression^42^, and in another study, miR-944 directly binds to CISH, mediating the cytokine-inducible Src homology 2-containing protein (CISH) downregulation and signal transducer and activator of transcription 3 (STAT3) phosphorylation upregulation, thereby promoting the progression of OSCC^43^. Distinct miR-944 expression patterns are observed in lung squamous cell carcinoma versus lung adenocarcinoma, and miR-944 modulates malignant phenotypes in NSCLC cells through SOCS4 suppression, thereby promoting oncogenic growth and invasion^44^. The upregulation of miR-944 serves as a negative prognostic marker in both cervical cancer and head-neck squamous cell carcinoma, where its overexpression is consistently associated with diminished survival rates, but its elevation is related to favorable prognosis in other cancers^45^.

In this study, our analysis discovered that circ_CDR1as, which might be a ceRNA for miR-944, was lowly expressed in PSCC. By further predicting the downstream mRNA targets of miRNAs, it was revealed that GATA6 performed downregulation in accordance with circ_CDR1as in tumors as the target of miR-944, whose expression showed a substantial positive correlation with circ_CDR1as. However, the involvement of miR-944 in PSCC is based on prediction and correlation rather than direct experimental evidence. Among the mRNAs positively associated with circ_CDR1as, ZEB1 was reported to be expressed in sarcomatoid samples of PSCC by immunohistochemistry, but no significant correlation was proven between ZEB1 expression and the presence of epithelial-mesenchymal transition (EMT) 46. There have also been reports of interactions between miR-944 and GATA6 in different kinds of cancers. For instance, miR-944 binds to GATA6 to block the EGF-induced EMT pathway, effectively suppressing the proliferation, invasion, and migration of colorectal cancer (CRC) cells^47^, ^48^. GATA6 is a transcription factor that contributes to the normal development of mesodermal and endodermal tissues in organs. The amplification or transcriptional upregulation of GATA6, as well as activation of the WNT signaling pathway, can improve the overall survival rate of human pancreatic ductal carcinoma (PDAC) at advanced malignant stages. In contrast, the absence of GATA6 expression, which is associated with basal-like characteristics of PDAC, tends to have a poor prognosis^49^. Currently, no studies have explored the mechanism of GATA6 in PSCC, but its critical role in squamous cell carcinoma at other sites has been investigated. For example, GATA6 is a key regulator of lung development. In lung squamous cell carcinoma, GATA6 has a binding site in the promoter region of plasminogen activator urokinase (PLAU), and GATA6 inhibits PLAU transcription by binding to PLAU promoter region, down-regulating PLAU expression, thereby inhibiting the cancer-promoting effect of PLAU^50^. A pancreatic cancer (PC) study using a pancreatic organoid model combined with multi-omics analysis found that GATA6 deficiency and Wnt deficiency synergizing with genetic or hypoxia-mediated KDM6A inactivation to promote squamous cell reprogramming, thereby enhancing the environmental adaptation of squamous cells, and then induced the development of pancreatic squamous cell carcinoma^51^. The loss of GATA6 expression in pancreatic squamous cell carcinoma compared with classical pancreatic ductal carcinoma suggested that the restoration of GATA6 may induce the transformation of pancreatic squamous cell carcinoma to classical pancreatic ductal carcinoma, thereby improving the chemosensitivity of the tumor treatment drug folfirinox^52^. Our data indicates that the low expression of circ_CDR1as, which may function as a sponge for the miR-944 and then increase the inhibitory effect of miR-944 on GATA6, may be the cause of the reduced expression of GATA6 in PSCC tissues. GATA6 deficiency in PSCC may similarly enhance the environmental adaptability of squamous cells by promoting squamous cell reprogramming, thereby contributing to the progression of PSCC.

In addition to circ_CDR1as, our study also found that other 10 circRNAs can play an important role in the occurrence and development of PSCC through different ceRNA networks. Although this study did not conduct in-depth analysis of other circRNAs, some of them have been explored in tumors. For example, hsa_circ_0086376 (circNFIB) is downregulated in breast cancer, which can encode a short peptide composed of 56 amino acids, and its downregulation can inhibit the growth and metastasis of breast cancer by reducing the synthesis of arachidonic acid^53^. And in intrahepatic cholangiocarcinoma, circNFIB inhibits tumor growth and metastasis by inhibiting MEK1/ERK signaling^54^. The has_circ_0008234 (circFOXP1) acts as a sponge for miR-370 to regulate PKLR, thereby promoting the Warburg effect in the progression of GBC^55^. In addition, has_circ_0008234 also plays a cancer-promoting role in other tumors through ceRNA network. For example, has_circ_0008234/miR-338-3p/ETS1 axis promotes colon cancer progression^56^; The has_circ_0008234/miR-204-5p/FGFR2 axis regulates the biological process of gallbladder cancer^57^; The has_circ_0001944 was found to be highly expressed in colorectal cancer samples and could promote the malignant progression of colorectal cancer by targeting miR-548b-3p^58^. The has_circ_0001610 was also found to play a pro-cancer role through different ceRNA networks in a variety of tumors, for example, hsa_circ_0001610/miR-139-5p/SLC7A11 can inhibit ferroptosis in breast cancer cells^59^; The hsa_circ_0001610/miR-1324/PTK6 axis can accelerate the malignant progression of prostate cancer^60^; The hsa_circ_0001610/miR-139-5p/ Cyclin B1 axis can attenuate the radiosensitivity of endometrial cancer^61^.

In this study, the tumor and adjacent samples of clinical PSCC patients were collected for circular RNA sequencing. Our aim was to explore the role of circular RNAs in PSCC progression via ceRNA networks, with the goal of identifying novel therapeutic targets and prognostic biomarkers for PSCC patients. However, there are still some limitations in this study. Initially, we attempted to use existing cell lines, such as Penl1, Penl2 and LM156^62^. However, despite submitting applications to other institutions and making attempts to establish primary cell cultures, we were ultimately unable to obtain viable PSCC cell lines for subsequent experimental validation due to inherent challenges, including tumor heterogeneity, clinical rarity, and consequent extreme difficulties in obtaining available tissue samples. Consequently, this study could not verify the effects of circ_CDR1as and GATA6 on PSCC cells by loss and gain of function experiments in vitro. Next, the clinical sample size for sequencing is small, so it is necessary to continue collecting clinical samples in the future to expand the sample size and improve clinical reliability.

## Conclusions

Our study identified a circRNA-miRNA-mRNA ceRNA network for PSCC, revealing possible molecular mechanisms by which key circRNA circ_CDR1as involved in the regulation of PSCC progression. The ceRNA network provides a novel perspective for elucidating the pathogenesis of PSCC and highlights circ_CDR1as and GATA6 as potential therapeutic targets for PSCC patients.

## Supplementary Material

Supplementary tables.

## Figures and Tables

**Table 1:** The clinical information of PSCC patients.

Patient	Age	T	N	M	Stage	Grade	p16	Tissue resource
Tumor 1	54	3	3	0	IV	G1	p16-	glans penis
Normal 1	74	1	—	0	—	G2	—	glans penis
Tumor 2 (Normal 2)	71	2	0	0	IIa	G1	p16+	glans penis
Tumor 3	69	—	—	—	—	G1	—	glans penis
Tumor 4 (Normal 3)	63	1b	1	0	IIIa	G2	p16+	glans penis
Tumor 5 (Normal 4)	76	3	3	0	IV	G2	p16-	glans penis
Tumor 6 (Normal 5)	67	1b	0	0	IIa	G1	p16-	glans penis

**Table 2:** Prediction of target miRNAs regulated by 41 known circRNAs

	circRNA	miRNA
starBase	27	293
circBank	41	1580
Circular RNA Interactome	41	304
intersection	11	20

**Table 3:** Prediction of target mRNAs regulated by 20 miRNAs

	miRNA	mRNA
miRDB	19	6199
miRTarBase	20	2213
TargetScan	20	14108
common targets	18	235
positively circRNA-related DE-mRNAs (r > 0.7, *p* < 0.05)	/	2457
Intersection	14	33

**Table 4:** Correlation analysis of circ_CDR1as with its predicted target mRNAs

circRNA	mRNA	r	*p* value	regulation
circ_CDR1as	GATA6	0.947	0.00001	positive
circ_CDR1as	SHISA6	0.915	0.00008	positive
circ_CDR1as	FAM189A1	0.333	0.31661	non-sig
circ_CDR1as	ZNF853	0.083	0.80931	non-sig
circ_CDR1as	PAX5	-0.264	0.43213	non-sig
circ_CDR1as	STRIP2	-0.351	0.28949	non-sig
circ_CDR1as	TSPAN13	-0.380	0.24956	non-sig
circ_CDR1as	HMGA1	-0.403	0.21918	non-sig
circ_CDR1as	OAS2	-0.427	0.19023	non-sig
circ_CDR1as	BCL11B	-0.442	0.17346	non-sig

## Abbreviations

PSCC: penile squamous cell carcinoma
ceRNA: competing endogenous RNA
DE-circRNAs: differentially expressed circRNAs
DE-mRNAs: differentially expressed mRNAs
miRNAs: microRNAs
lncRNAs: long non-coding RNAs
circRNAs: circular RNAs
MREs: miRNA response elements
TNBC: triple-negative breast cancer
CRC: colorectal cancer
FDR: False Discovery Rate
RPM: Reads Per Million mapped reads
CDR1: cerebellar degeneration-related protein 1
OSCC: oral squamous cell carcinoma
NSCLC: non-small cell lung cancer
HCC: hepatocellular carcinoma
CISH: cytokine-inducible Src homology 2-containing protein
STAT3: signal transducer and activator of transcription 3
EMT: epithelial-mesenchymal transition
PDAC: pancreatic ductal carcinoma
